# 23-Hydroxybetulinic acid attenuates 5-fluorouracil resistance of colorectal cancer by modulating M2 macrophage polarization via STAT6 signaling

**DOI:** 10.1007/s00262-024-03662-0

**Published:** 2024-03-30

**Authors:** Zeping Fan, Yaru Cui, Lanying Chen, Peng Liu, Wenbin Duan

**Affiliations:** 1 School of Pharmacy, Jiangxi University of Chinese Medicine, Nanchang, 330006 Jiangxi China; 2 National Pharmaceutical Engineering Center for Solid Preparation of Chinese Herbal Medicine, Jiangxi University of Chinese Medicine, Nanchang, 330006 Jiangxi China; 3 Key Laboratory for Evaluation on Anti-Tumor Effect of Chinese Medicine by Strengthening Body Resistance to Eliminate Pathogenic Factors, Nanchang, 330006 Jiangxi China

**Keywords:** 23-Hydroxybetulinic acid, Macrophage polarization, STAT6, Colorectal cancer, 5-fluorouracil, Chemoresistance

## Abstract

Macrophage polarization is closely associated with the inflammatory processes involved in the development and chemoresistance of colorectal cancer (CRC). M2 macrophages, the predominant subtype of tumor-associated macrophages (TAMs) in a wide variety of malignancies, have been demonstrated to promote the resistance of CRC to multiple chemotherapeutic drugs, such as 5-fluorouracil (5-FU). In our study, we investigated the potential of 23-Hydroxybetulinic Acid (23-HBA), a significant active component of *Pulsatilla chinensis* (*P. chinensis*), to inhibit the polarization of M2 macrophages induced by IL-4. Our results showed that 23-HBA reduced the expression of M2 specific marker CD206, while downregulating the mRNA levels of M2 related genes (CD206, Arg1, IL-10, and CCL2). Additionally, 23-HBA effectively attenuated the inhibitory effects of the conditioned medium from M2 macrophages on apoptosis in colorectal cancer SW480 cells. Mechanistically, 23-HBA prevented the phosphorylation and nuclear translocation of the STAT6 protein, resulting in the inhibition of IL-10 release in M2 macrophages. Moreover, it interfered with the activation of the IL-10/STAT3/Bcl-2 signaling pathway in SW480 cells, ultimately reducing M2 macrophage-induced resistance to 5-FU. Importantly, depleting STAT6 expression in macrophages abolished the suppressive effect of 23-HBA on M2 macrophage polarization, while also eliminating its ability to decrease M2 macrophage-induced 5-FU resistance in cancer cells. Furthermore, 23-HBA significantly diminished the proportion of M2 macrophages in the tumor tissues of colorectal cancer mice, simultaneously enhancing the anti-cancer efficacy of 5-FU. The findings presented in this study highlight the capacity of 23-HBA to inhibit M2 macrophage polarization, a process that contributes to reduced 5-FU resistance in colorectal cancer.

**Supplementary Information:**

The online version contains supplementary material available at 10.1007/s00262-024-03662-0.

## Introduction

Colorectal cancer (CRC) is among the most lethal malignancies globally, characterized by a dismal prognosis and low survival rates [1]. The primary strategy for treating colorectal cancer (CRC) is systemic chemotherapy based on 5-fluorouracil (5-FU), yet it is constrained by drug resistance [2]. Recent research indicates that the therapeutic response of tumor cells is influenced not solely by their genomic aberrations but also by the tumor microenvironment (TME), particularly the inflammatory processes initiated by immune cells [3–6]. Tumor-associated macrophages (TAMs), also referred to as M2 macrophages, serve as the primary immune cells that infiltrate the tumor [7]. Numerous studies suggest that M2 macrophages facilitate the initiation and dissemination of tumor cells while also enhancing the tumor cells’ capacity to withstand cytotoxic chemotherapy [7–10]. Targeting TAMs has been demonstrated to suppress tumor progression and decrease chemoresistance [9, 11–14].

STAT6, a signal transducer and activator of transcription 6, has shown promise as a powerful target for anti-cancer medications [15, 16]. Clinical evidence reveals a positive correlation between high levels of STAT6 expression and poor clinical outcomes in colorectal cancer patients [17]. A previous study demonstrated that STAT6-deficient mice displayed fewer and smaller colorectal tumors compared to wild-type mice [18]. Additionally, the STAT6 phosphorylation inhibitor AS1517499 significantly slowed the growth of colorectal tumors in mice when administered in combination with 5-FU [19]. These results indicate that the activation of STAT6 may contribute to the growth and drug resistance of colorectal cancer. STAT6 signaling is primarily activated by IL-4 and IL-13, which are critical cytokines for M2 macrophage polarization [20]. When IL-4 or IL-13 binds to the receptor, Janus kinase (JAK) is phosphorylated. This results in phosphorylation of tyrosine residues in the cytoplasmic region of the receptor, which provide multiple binding sites for the STAT6 molecule [21, 22]. Once activated, STAT6 dissociates from the receptor to form a homodimer and enters the nucleus, where it binds to certain accessible DNA sequences, initiating the transcription of related genes and leading to the polarization of M2 macrophages [21, 22]. Consequently, pharmacological inhibition of the STAT6 pathway emerges as a feasible strategy to regulate macrophage polarization, potentially impeding tumor progression and reducing chemoresistance in colorectal cancer.

Recently, there has been a growing interest in integrating natural bioactive ingredients with chemotherapeutic medications for cancer treatment [23]. This treatment strategy focuses on leveraging multiple anti-cancer mechanisms to enhance the inhibition of carcinogenesis, mitigate drug resistance, and alleviate chemotherapy-related side effects [24]. *Pulsatilla chinensis* (Bunge) Regel, a well-known traditional Chinese medicine, is recognized for its “blood-cooling” and detoxification properties [25]. Among its significant active compounds is 23-hydroxybetulinic acid (23-HBA), a pentacyclic triterpenoid extracted from the root of *P. chinensis*. Previous studies have suggested that, besides inducing apoptosis in cancer cells, 23-HBA may also augment the efficacy of certain anti-tumor medications [26, 27]. However, the majority of research on 23-HBA in cancer has focused on its direct effects on cancer cells, with limited exploration of its influence on the tumor microenvironment, particularly regarding macrophages. In this study, we investigated whether 23-HBA could inhibit M2 macrophage polarization and subsequently attenuate M2 macrophage-mediated 5-FU resistance of colorectal cancer. The findings demonstrated that 23-HBA can attenuate 5-FU resistance in colorectal cancer by inhibiting M2 macrophage polarization via STAT6 signaling.

## Materials and methods

### Reagents

23-Hydroxybetulinic Acid (Push Biotechnology Co., Ltd., Chengdu, China) was dissolved in DMSO at a concentration of 10 mg/mL. 5-FU was purchased from Solarbio Biotechnology Co., Ltd. (Beijing, China). PMA was purchased from Sigma (MO, USA). Recombinant Human IL-4 was purchased from PeproTech (Rocky Hill, NJ, USA).

### Cell lines and mice

THP-1 and SW480 cell lines were obtained from the Shanghai Cell Collection, Chinese Academy of Sciences. The cells were cultured in RPMI-1640 medium containing 10% FBS and incubated in a 5% CO2 humidified incubator. Male BALB/c mice (6–8 weeks old, weighing 20 ± 2 g) were purchased from Hunan SJA Laboratory Animal Co., Ltd. (Changsha, China). The mice were placed in a constant temperature environment (23 ± 2 °C), light/dark cycle for 12 h, and were free to eat and drink.

### Macrophage polarization and conditioned medium preparation

THP-1 cells incubated with PMA (150 nM) were seeded in 6-well plates at a density of 2 × 10^6 cells per well for 48 h to generate M0 macrophages. M0 macrophages were treated with IL-4 (20 ng/mL) with or without 23-HBA for 48 h to induce M2 macrophages. The cells were then used for further studies. Different types of macrophages were cultured in 2 mL of serum-free medium for an additional 72 h. The cell supernatants were collected, centrifuged, and mixed with fresh medium at a ratio of 30:70 to generate conditioned medium (CM) [28].

### Cell viability assay

THP-1 cells incubated with PMA were seeded in 96-well plates at a density of 8 × 10^3 cells per well for 48 h. Subsequently, the cells were exposed to varying concentrations of 23-HBA for an additional 48 h. Afterward, 10 µL of CCK-8 solution was added to each well, and the plates were incubated in an incubator for 1 h. Finally, the OD value of each well was measured at 450 nm using a molecular device (Infinite M200 PRO, Switzerland). Similarly, SW480 cells were seeded in 96-well plates for 24 h. The culture medium was replaced with conditioned medium from M0 macrophages and M2 macrophages, respectively. Varying concentrations of 5-FU were added simultaneously, and after 48 h, the OD value of each well was measured. The half inhibitory concentration (IC50) was calculated using GraphPad Prism as the subsequent 5-FU concentration. Following the same method, SW480 cells were seeded in 96-well plates, and the culture medium was substituted with conditioned medium from M0 macrophages, M2 macrophages, and M2 macrophages intervened with 23-HBA, respectively. Simultaneously add 5-FU (45 µM) and measure the OD value of each well after 48 h.

### Real-time PCR assay (RT-PCR)

Macrophages or tumor tissues were used to extract total RNA with Trizol, and the Hifair® III 1st Strand cDNA Synthesis Kit (Yeasen Biotech Co., Ltd, Shanghai, China) was used to synthesize cDNA. Primers for human CD206, Arg1, IL-10, and CCL2, as well as for mouse IL-10, were purchased from Genscript Biotech (Beijing, China). The PCR assay was performed on an Applied Biosystems 7500 instrument in accordance with the manufacturer's instructions from Foster, CA, USA. The information was standardized to GAPDH and examined using 2^−△△Ct^ method. Primer sequences are listed in Table S1.

### Molecular docking

The target proteins’ crystal structures were obtained from the PDB database (https://www.rcsb.org/) and then imported into PyMOL software. PyMOL software was utilized to eliminate water molecules and heteromolecules. The drug small molecules were obtained from the PubChem database in MOL2 format. Molecular docking between the target proteins and drug small molecules was performed using the Autodock 4 software program. The binding energy resulting from the molecular docking experiments was used as a docking score to assess the protein–ligand binding potential.

### Immunofluorescence of cells

Macrophages were fixed with 4% paraformaldehyde for 10 min, followed by permeabilization with 0.2% Triton X-100 for 15 min, and blocked using 5% BSA for 1 h. Next, the cells were incubated with primary antibodies anti-p-STAT6 (AF3301, Affinity, Cincinnati, OH, USA, 1:100) and anti-CD206 (60143-1, Proteintech, Wuhan, China, 1:100) at 4 °C overnight. The cells were washed 3 times with PBS and then incubated with secondary antibodies Alexa Fluor-594 goat anti-rabbit IgG (A11012, Invitrogen, Carlsbad, CA, USA, 1:1000) and Alexa Fluor-488 goat anti-mouse IgG (A11001, Invitrogen, 1:1000) for 1 h at room temperature. Following this, the cells underwent staining with DAPI to visualize the nuclei. Finally, the images were acquired using a confocal microscope (Leica TCS SP8, Germany).

### Flow cytometry

The M2-specific marker CD206 was determined through flow cytometry. THP-1 macrophages were differentiated using a process that has been previously described. The collected cells were incubated with APC-conjugated anti-human CD206 (321110, Biolegend, San Diego, CA, USA) for 30 min at 4 °C. Afterward, the cells were cleansed and suspended in PBS before being subjected to flow cytometry analysis using a Gallios Flow Cytometer (Beckman Coulter, Brea, CA, USA).

### Small interfering RNA (siRNA) transfection

Small interfering RNA (siRNA) was purchased from Rib Bio Biotechnology Co., Ltd. (Guangzhou, China) and used for STAT6 gene knockdown. THP-1 cells were incubated with PMA for 48 h before siRNA was transfected into cells using Lipofectamine 3000 (Thermo Fisher, Waltham, MA, USA). After 48 h of transfection, the cells were treated with IL-4 (20 ng/mL) with or without 23-HBA for 48 h and then used for further studies.

### Western blot analysis

Proteins were collected, quantified with a BCA kit, and boiled samples (20 μg) were separated via SDS-PAGE, then transferred to a PVDF membrane. The membrane was blocked with 5% skim milk for 2 h and then incubated with primary antibodies, including anti-p-STAT6 (ab263947, Abcam, Cambridge, UK, 1:1000), anti-STAT6 (ab32520, Abcam, 1:1000), anti-p-JAK2 (ab32101, Abcam, 1:1000), anti-JAK2 (ab108596, Abcam, 1:1000), anti-p-STAT3 (ab76315, Abcam, 1:1000), anti-STAT3 (ab68153, Abcam, 1:1000), anti-Bcl-2 (ab32124, Abcam, 1:1000) and anti-β-tubulin (TA503129, OriGene, Rockville, MD, USA, 1:1000) at 4 ℃ overnight. Next, the membrane was incubated with an HRP-conjugated secondary antibodies for 1 h and proteins were visualized using the ECL Western Blotting Detection System (ChemiDoc XRS, Bio-Rad, Hercules, CA, USA).

### Cell apoptosis analysis

SW480 cells were seeded in 6-well plates at a density of 5 × 10^4 cells per well for 24 h. The culture medium was then replaced with conditioned medium from M0 macrophages, M2 macrophages, and M2 macrophages intervened with 23-HBA, respectively. 5-FU (45 µM) was simultaneously added. Apoptosis analysis was conducted after 48 h of incubation using the FITC Annexin V apoptosis detection kit (MultiSciences Biotech, Hangzhou, China).

### Analysis of cytokine profile in conditioned medium

The Human Cytokine Antibody Arrays Kit from RayBiotech (Norcross, GA, U.S.) was employed to detect cytokines in the conditioned medium. In short, the arrays were initially obstructed using a blocking buffer and subsequently exposed to conditioned medium, a biotin-conjugated antibody, and a HPR-conjugated secondary antibody for 2 h each. The arrays were visualized using the ECL Western Blotting Detection System.

### Enzyme-linked immunosorbent assay (ELISA)

The macrophages conditioned media were prepared according to the above method. Human quantitative IL-10 ELISA kits (MultiSciences Biotech, Hangzhou, China) were used to measure IL-10 levels in the conditioned medium following the manufacturer’s protocol.

### In vivo experiments

CT26 cells (2 × 10^6^) suspended in cold PBS were subcutaneously injected into the right armpits of BALB/c mice. After 24 h of inoculation, the mice were randomly divided into seven groups (*n* = 6/group) and treated as follows: model group (saline administered intraperitoneally daily), 5-FU group (25 mg/kg 5-FU administered intraperitoneally once every 3 days), 23-HBA group (7.5 and 15 mg/kg 23-HBA administered intraperitoneally daily), 23-HBA combined with 5-FU administration groups (7.5 and 15 mg/kg 23-HBA administered intraperitoneally daily, and 25 mg/kg 5-FU administered intraperitoneally once every 3 days), and an additional 6 mice were employed as a normal control group. The body weight of each mouse was monitored every 2 days. The mice were anesthetized and euthanized with pentobarbital sodium (90 mg/kg) after 24 h of final drug or saline administration, and the tumors were removed.

### Tissue immunohistochemistry

Tumor tissue slices with a thickness of 5 μm were immunostained. The sections were heat-induced in Tris–EDTA solution to recover antigen, then treated with 3% hydrogen peroxide for 10 min and preincubated with 10% normal goat serum for 10 min. Subsequently, the sections were incubated with the primary antibodies anti-p-STAT6 (AF3301, Affinity, 1:500) and anti-CD206 (60143-1, Proteintech, 1:200) at 4 °C overnight. The following day, the sections were thoroughly washed with PBS and incubated with secondary antibodies at room temperature for 10 min. The reaction was visualized using 3,3-diaminobenzidine (DAB). and the sections were subsequently counter-stained with hematoxylin, dehydrated, and fixed. Finally, the sections were observed and photographed using a microscope and analyzad by ImageJ software.

### Statistical analysis

Statistical analysis was performed using GraphPad Prism 9.0 software. Data are presented as mean ± standard deviation (SD). The differences between two groups were compared using Student’s *t* test. One-way ANOVA analysis was used for comparison among groups. The *p* < 0.05 were considered statistically significant.

## Results

### 23-HBA suppressed IL-4-induced M2 macrophage polarization in vitro

**Fig. 1:**
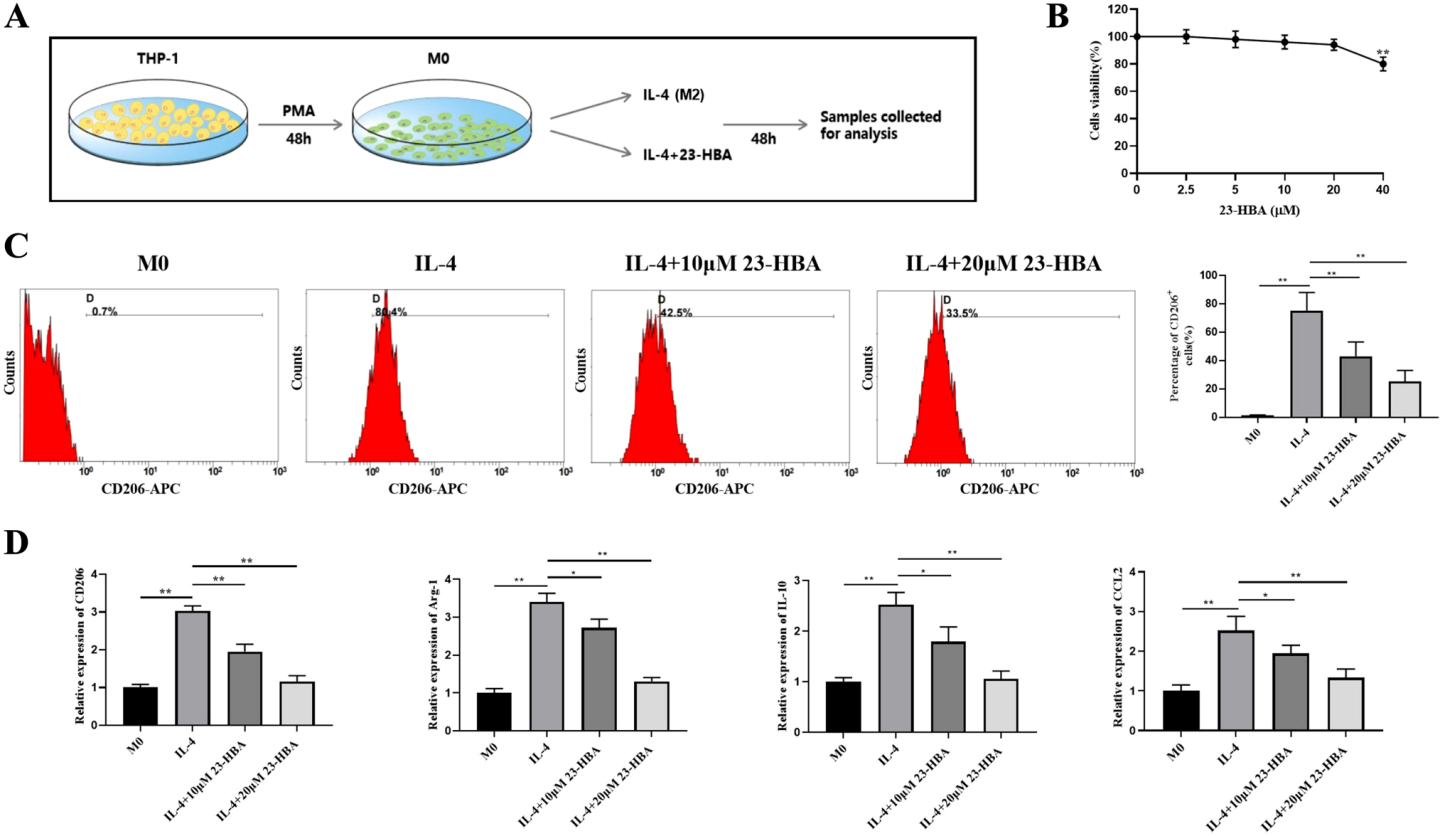
23-HBA inhibited M2 macrophage polarization induced by IL-4. **A** Schematic diagram of experiment. **B** Analysis of the administration of 23-HBA (2.5, 5, 10, 20, 40 μM) on cell viability of M0 macrophages for 48 h. **C, D** M0 macrophages were treated with IL-4 (20 ng/mL) with or without 23-HBA (10, 20 μM) for 48 h. These treated cells were collected for the following analyses. **C** The expression of CD206 was analyzed by flow cytometry. **D** RT-PCR was performed to analyze the mRNA levels of CD206, Arg-1, IL-10, and CCL2. The GAPDH gene was used as internal control. Data were presented as mean ± SD (*n* = 3). Statistical significance: *p < 0.05, **p < 0.01

As widely acknowledged, M2 macrophage polarization plays a pivotal role in the development of colorectal cancer [29]. Therefore, we explored the potential of 23-HBA to influence macrophage polarization (Fig. 1A). First, we evaluated the cytotoxicity of 23-HBA on THP-1-derived macrophages (M0 macrophages) to determine the appropriate concentration. As shown in Fig. 1B, 23-HBA is safe for M0 macrophages in the concentration range of 0–20 μM. So, we selected 10 and 20 μM for in vitro experiments. Next, we employed flow cytometry to measure the expression of CD206, a specific marker for M2 macrophages. The results indicated that IL-4 strongly stimulated CD206 expression; whereas, 23-HBA significantly inhibited its expression in a concentration-dependent manner (Fig. 1C). To further confirm the effect of 23-HBA on M2 polarization, the mRNA levels of M2 macrophage-associated genes was assessed by RT-PCR. The findings demonstrated a significant upregulation of CD206, Arg-1, IL-10, and CCL2 expression in IL-4-induced macrophages, which was considerably decreased in a concentration-dependent manner by 23-HBA (Fig. 1D). Taken together, these results suggest that 23-HBA effectively inhibited IL-4-induced M2 macrophage polarization in vitro.

### 23-HBA impaired M2 macrophage polarization by preventing STAT6 phosphorylation and nuclear translocation

**Fig. 2:**
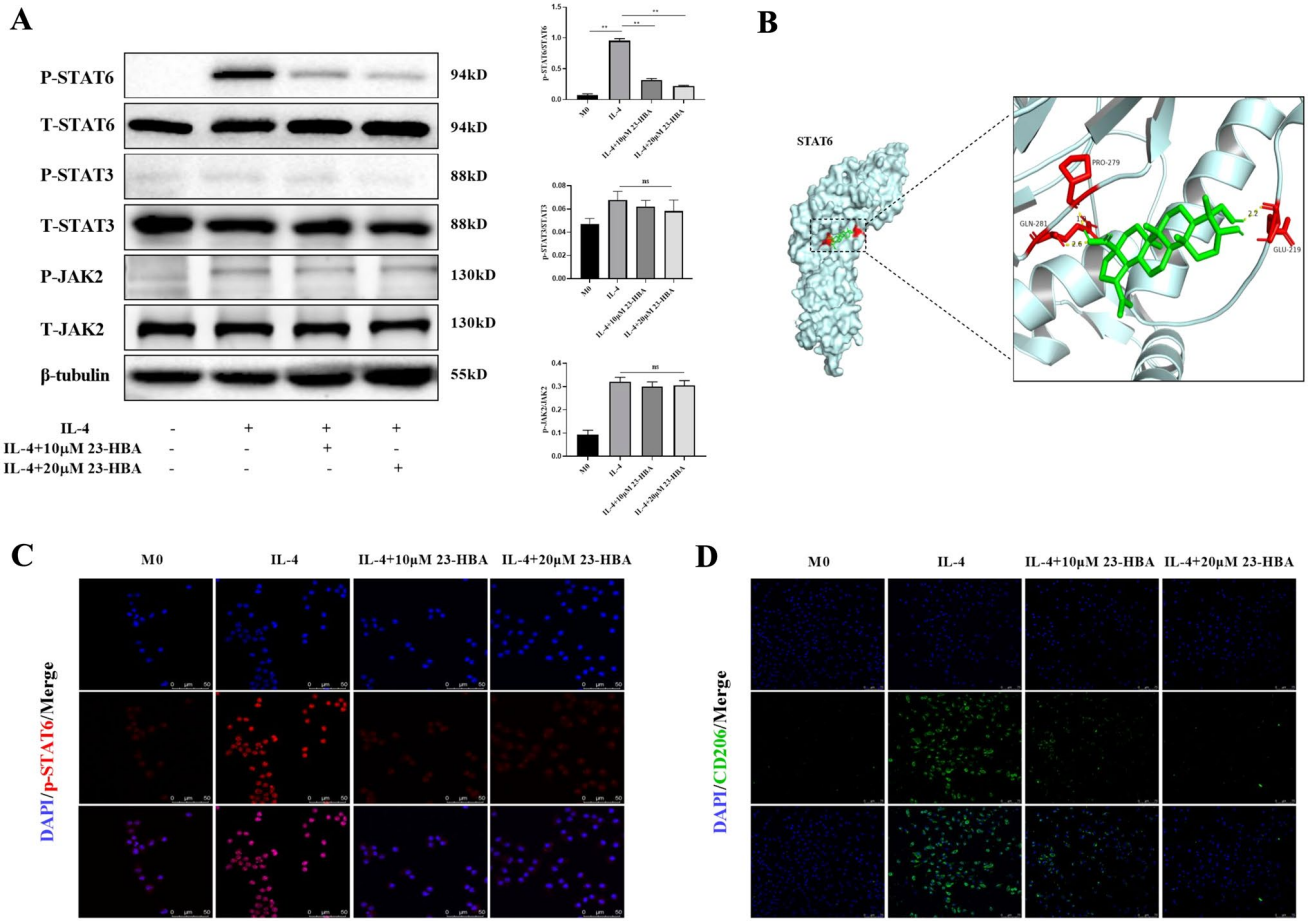
23-HBA inhibited IL-4-induced phosphorylation and nuclear translocation of STAT6 in macrophages. M0 macrophages were treated with IL-4 (20 ng/mL) with or without 23-HBA (10, 20 μM) for 48 h. These treated cells were collected for the following analyses. **A** Relative protein levels of p-STAT6/STAT6, p-STAT3/STAT3, and p-JAK2/JAK2 were determined by western blot. β-tubulin was used as internal control. **B** Docking simulation results of 23-HBA and STAT6. **C** The nuclear translocation of STAT6 was detected by immunofluorescence. Bars represent 50 μm. **D** The expression of M2 marker CD206 was detected by immunofluorescence. Bars represent 75 μm. Data were presented as mean ± SD (*n* = 3). Statistical significance: *p < 0.05, **p < 0.01, *n.s* no significance

Recent research have highlighted the importance of the JAK2/STAT6 signaling pathway in M2 macrophage polarization [30, 31]. Given the context of 23-HBA-mediated regulation of M2 macrophage polarization, we conducted an investigation into this signaling pathway. First, phosphorylation levels of JAK2 and STAT6 proteins were determined using western blot. The results displayed a notable increase in the phosphorylation of both proteins in macrophages stimulated with IL-4 (Fig. 2A). However, the presence of 23-HBA significantly reduced the level of STAT6 protein phosphorylation in a concentration-dependent manner, with no impact on JAK2 protein phosphorylation (Fig. 2A). Additionally, we observed a slight increase in STAT3 protein phosphorylation in IL-4-stimulated macrophages, which was partially inhibited by 23-HBA at a concentration of 20 µM, but this effect did not reach statistical significance (Fig. 2A). Molecular docking was utilized to determine the STAT6 and 23-HBA binding affinity. The findings demonstrate the stability of the 23-HBA-STAT6 complex, as indicated by a docking score of − 7.04 (Fig. 2B). The STAT6 protein binding site involved multiple interactions with the 23-HBA molecule. Specifically, Glu 219 residue of the STAT6 protein formed a hydrogen bond with a hydrogen (H) atom on the 23-HBA molecule. Furthermore, the STAT6 protein’s Gln 281 and Pro 279 residues established hydrogen bonds with an oxygen (O) atom on the 23-HBA molecule (Fig. 2B). Immunofluorescence assay demonstrated that 23-HBA inhibited IL-4-induced STAT6 nuclear translocation (Fig. 2C) and CD206 expression (Fig. 2D). Collectively, these data illustrated that 23-HBA blocked M2 macrophage polarization by preventing STAT6 phosphorylation and nuclear translocation.

**Fig. 3:**
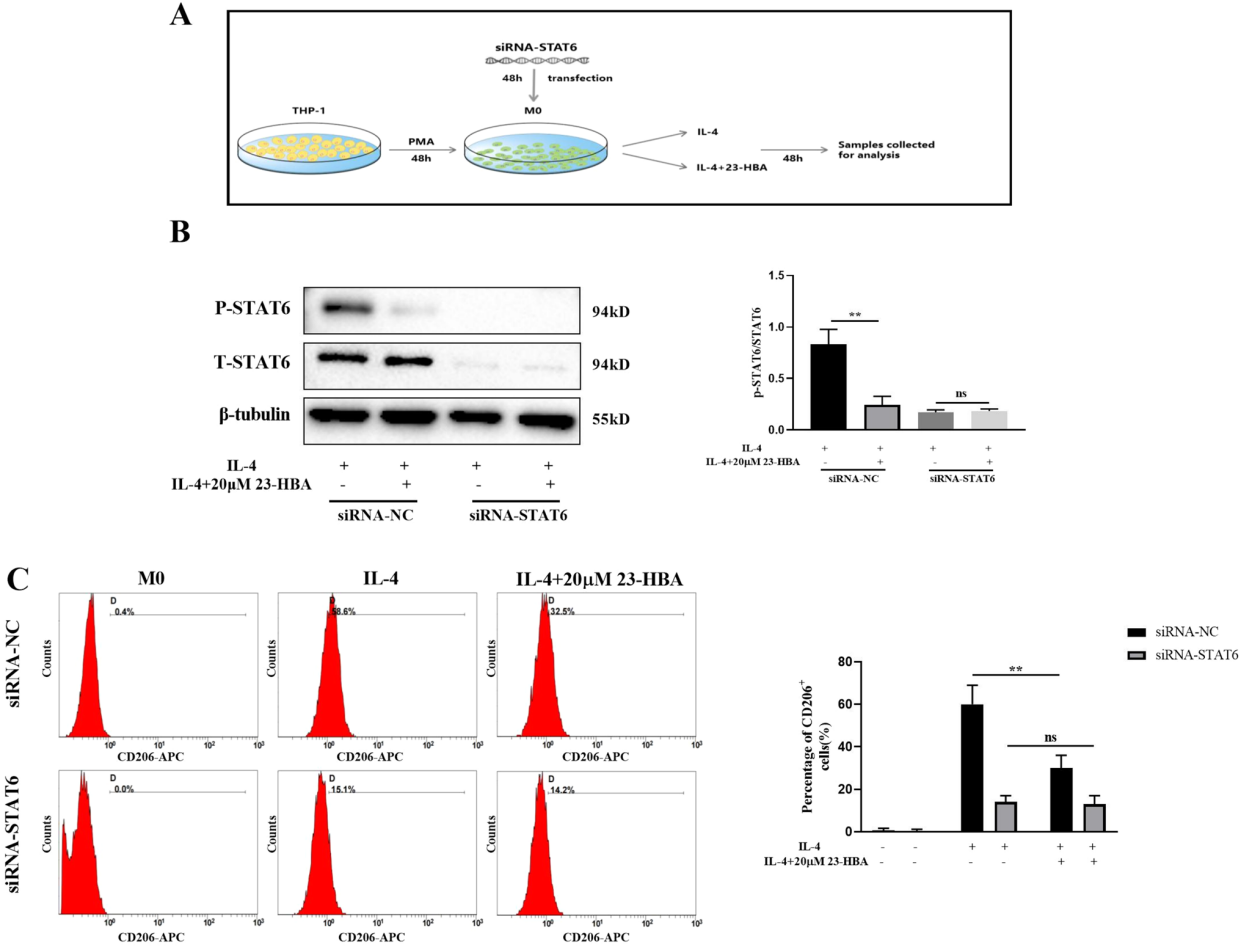
23-HBA inhibited M2 macrophage polarization in a STAT6-dependent manner. **A** Schematic diagram of experiment. M0 macrophages were treated with siRNA-NC or siRNA-STAT6 for 48 h, and then treated with IL-4 (20 ng/mL) with or without 23-HBA (20 μM) for 48 h. These treated cells were collected for the following analyses. **B** Relative protein levels of p-STAT6/STAT6 were determined by western blot. β-tubulin was used as internal control. **C** The expression of CD206 was analyzed by flow cytometry. Data were presented as mean ± SD (*n* = 3). Statistical significance: *p < 0.05, **p < 0.01, *n.s* no significance

To investigate the relationship between STAT6 signaling and the inhibitory impact of 23-HBA on M2 polarization, we employed STAT6 small interfering RNA (siRNA) to suppress STAT6 expression in M0 macrophages (Fig. 3A). The findings showed that the phosphorylation of STAT6 protein was absent in macrophages transfected with siRNA-STAT6 after stimulation with IL-4 and/or 23-HBA (20 µM) (Fig. 3B). Simultaneously, flow cytometry was utilized to identify the M2 macrophage specific marker CD206. The results suggested that the inhibitory effect of 23-HBA on CD206 was impeded following STAT6 knockdown, indicating a compromised ability of 23-HBA to inhibit the polarization of M2 macrophages (Fig. 3C). Taken together, these findings demonstrated that 23-HBA effectively suppressed the polarization of M2 macrophages induced by IL-4 through its specific interaction with STAT6.

### 23-HBA attenuated M2 macrophage-mediated 5-FU resistance of SW480 cells

**Fig. 4:**
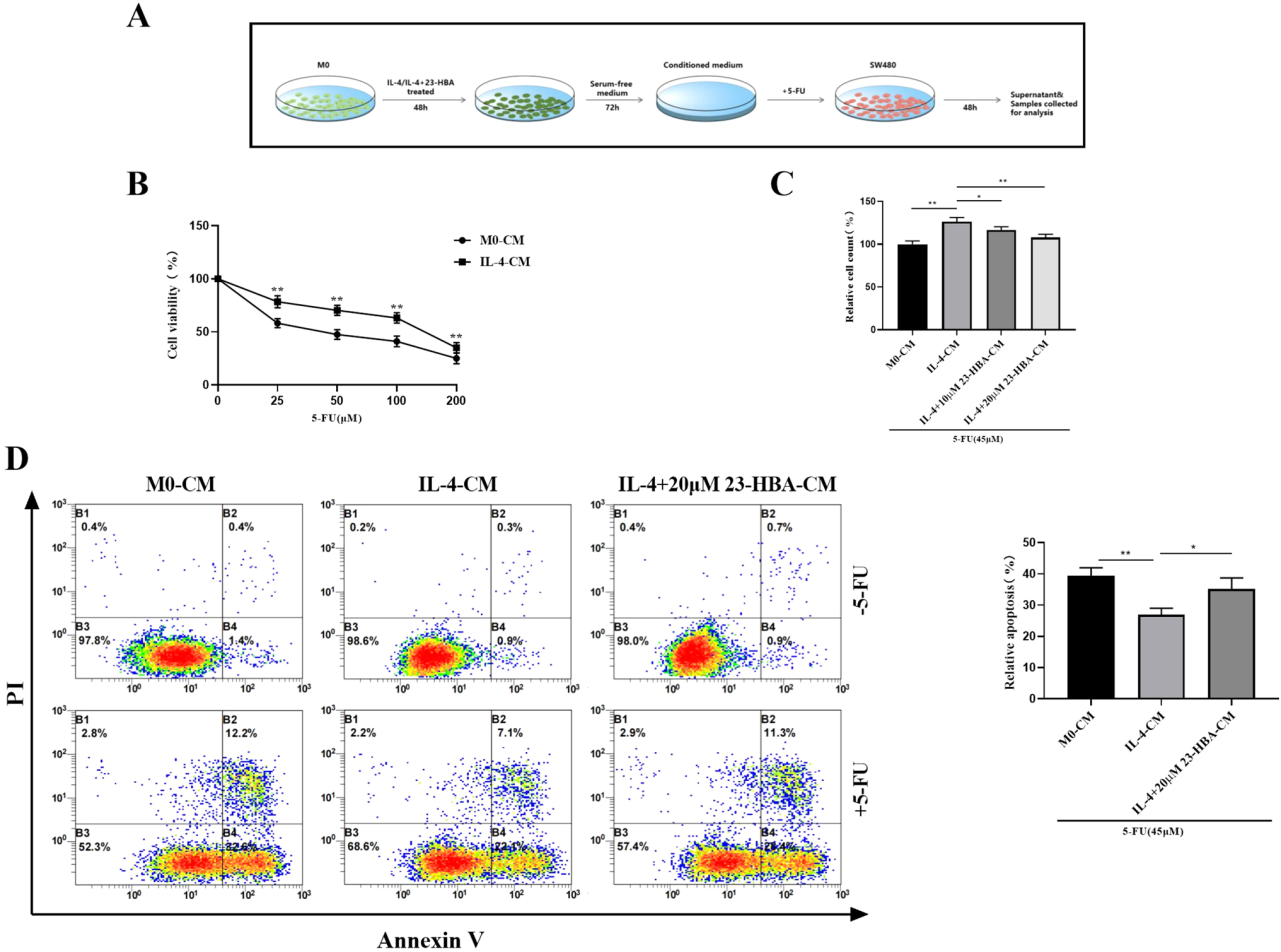
23-HBA attenuated M2 macrophage-mediated 5-FU resistance of SW480 cells. **A** Schematic diagram of experiment. **B** Dose–response curves showing the sensitivity of SW480 cells to 5-FU in M0-CM and IL-4-CM. **C** The CCK8 assay was used to measure the relative number of SW480 cells treated with 5-FU (45 µM) for 48 h in M0-CM, IL-4-CM, and IL-4+(10, 20 µM) 23-HBA-CM. **D** SW480 cells were treated with or without 5-FU (45 µM) in M0-CM, IL-4-CM, and IL-4+20 µM 23-HBA-CM for 48 h, and the percentage of apoptotic cells was analyzed by flow cytometry. Data were presented as mean ± SD (*n* = 3). Statistical significance: *p < 0.05, **p < 0.01

Colorectal cancer cells were cultivated using three different conditions: naive macrophages-conditioned medium (M0-CM), M0 macrophages treated with IL-4-conditioned medium (IL-4-CM), and M0 macrophages treated with both IL-4 and 23-HBA-conditioned medium (IL-4+23-HBA-CM). To construct dose–response curves in both M0-CM and IL-4-CM, SW480 cells were treated with incremental concentrations of 5-FU. As shown in Fig. 4B, the IC50 of 5-FU was 45 µM when SW480 cells were incubated with M0-CM. In contrast, when incubated with IL-4-CM, the IC50 increased to 140 µM, demonstrating that M2 macrophages significantly induced resistance of SW480 cells to 5-FU by threefold.

Next, we analyzed the relative numbers of SW480 cells treated with 5-FU (45 µM) for 48 h in the three different conditioned media. In comparison with cells incubated with IL-4-CM, a significant decrease in SW480 cell number was observed when exposed to IL-4+23-HBA-CM (Fig. 4C). Furthermore, flow cytometry analysis revealed that IL-4+23-HBA-CM greatly increased the percentage of apoptotic cells treated with 5-FU (45 µM) compared to IL-4-CM (Fig. 4D). Overall, these findings indicate that 23-HBA can attenuate M2 macrophage-mediated 5-FU resistance of SW480 cells.

**Fig. 5:**
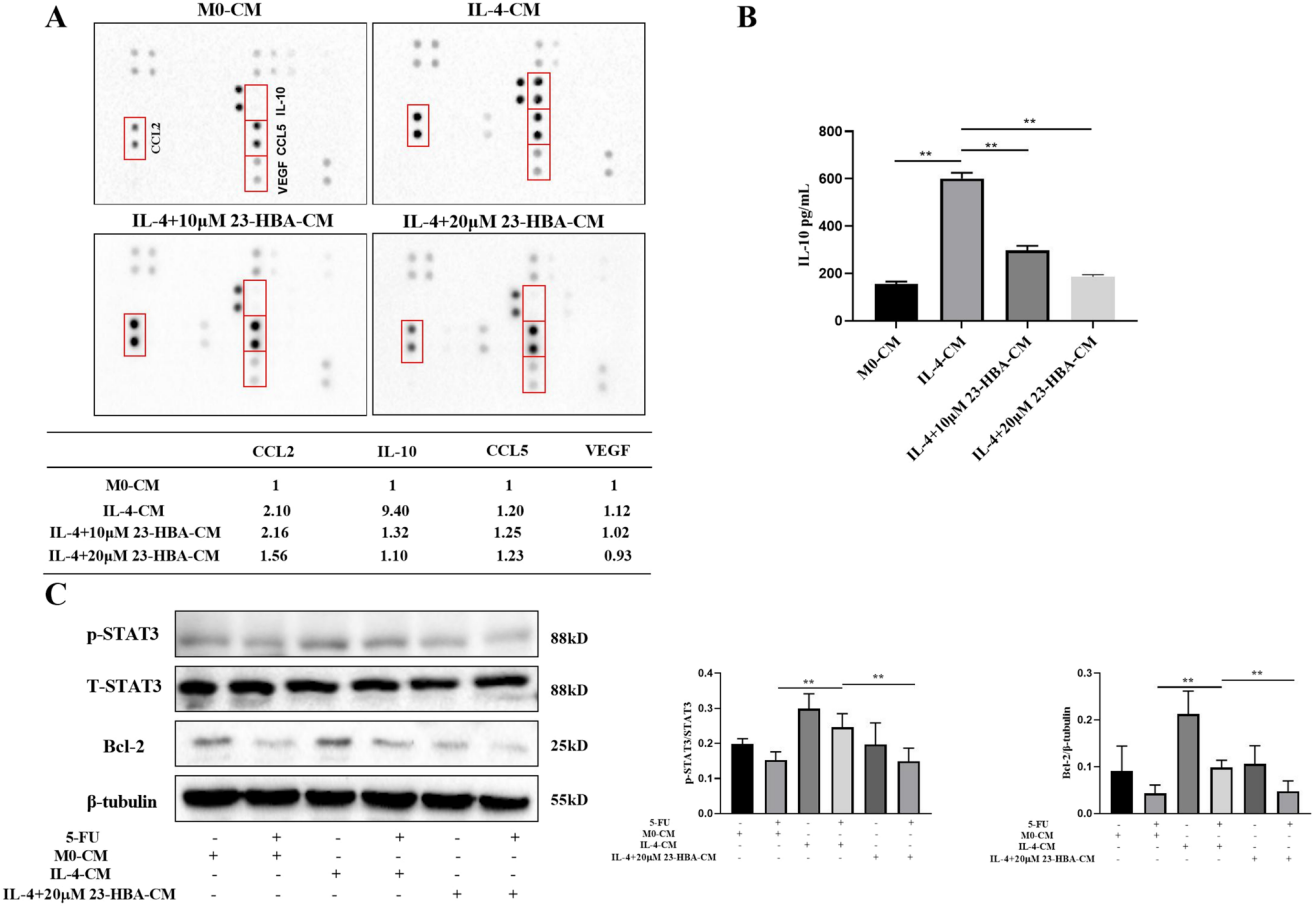
23-HBA reversed M2 macrophage-mediated signal response of SW480 cells. **A** Cytokine array analysis of the conditioned medium from the macrophages. The table in the bottom summarizes the relative signal intensity of the indicated cytokines. **B** IL-10 levels in M0-CM, IL-4-CM, and IL-4+(10, 20 µM) 23-HBA-CM were determined by ELISA. **C** SW480 cells were treated with or without 5-FU (45 µM) in M0-CM, IL-4-CM, and IL-4+20 µM 23-HBA-CM for 48 h, and the relative protein levels of p-STAT3/STAT3 and the expression of Bcl-2 protein were analyzed by western Blot. β-tubulin was used as internal control. Data were presented as mean ± SD (*n* = 3). Statistical significance: *p < 0.05, **p < 0.01

Considering the crucial role of cytokines in mediating signaling transduction between different cell types in the TME, we hypothesized that IL-4-induced M2 macrophages contribute to chemoresistance by secreting certain cytokines. To this end, we performed an analysis using a cytokine array and observed an increase in the levels of four cytokines (CCL2, IL-10, CCL5, and VEGF) in the IL-4-CM compared to the M0-CM (Fig. 5A). Among these cytokines, IL-10 exhibited the highest levels of upregulation and abundance (Fig. 5A). Moreover, administration of 23-HBA led to a decrease in CCL2, IL-10, and VEGF, with IL-10 exhibiting the most notable decline (Fig. 5A). These findings were further confirmed by an ELISA assay, which demonstrated a significant increase in IL-10 levels in the IL-4-CM and subsequent reversal by 23-HBA (Fig. 5B). Consequently, IL-10 was selected for further analysis.

To further explore the effect of 23-HBA inhibition of IL-10 release from M2 macrophages on chemotherapy resistance in colorectal cancer, we conducted an analysis of STAT3 and Bcl-2, both of which serve as key mediators within the IL-10 signaling pathway. The results indicated that SW480 cells treated with 5-FU (45 µM) showed a significant increase in the phosphorylation level of STAT3 protein and Bcl-2 expression when exposed to IL-4-CM relative to M0-CM (Fig. 5C). However, there was a significant reduction in the phosphorylation level of STAT3 protein and Bcl-2 expression after exposure to IL-4+23-HBA-CM (Fig. 5C). Based on the above results, we hypothesized that the downregulation of IL-10 derived from M2 macrophages by 23-HBA inhibited the subsequent activation of the IL-10/STAT3/Bcl-2 signaling pathway in SW480 cells, thereby reducing the resistance of SW480 cells to 5-FU.

### 23-HBA reduced M2 macrophage-induced 5-FU resistance of SW480 cells in a STAT6-dependent manner

**Fig. 6:**
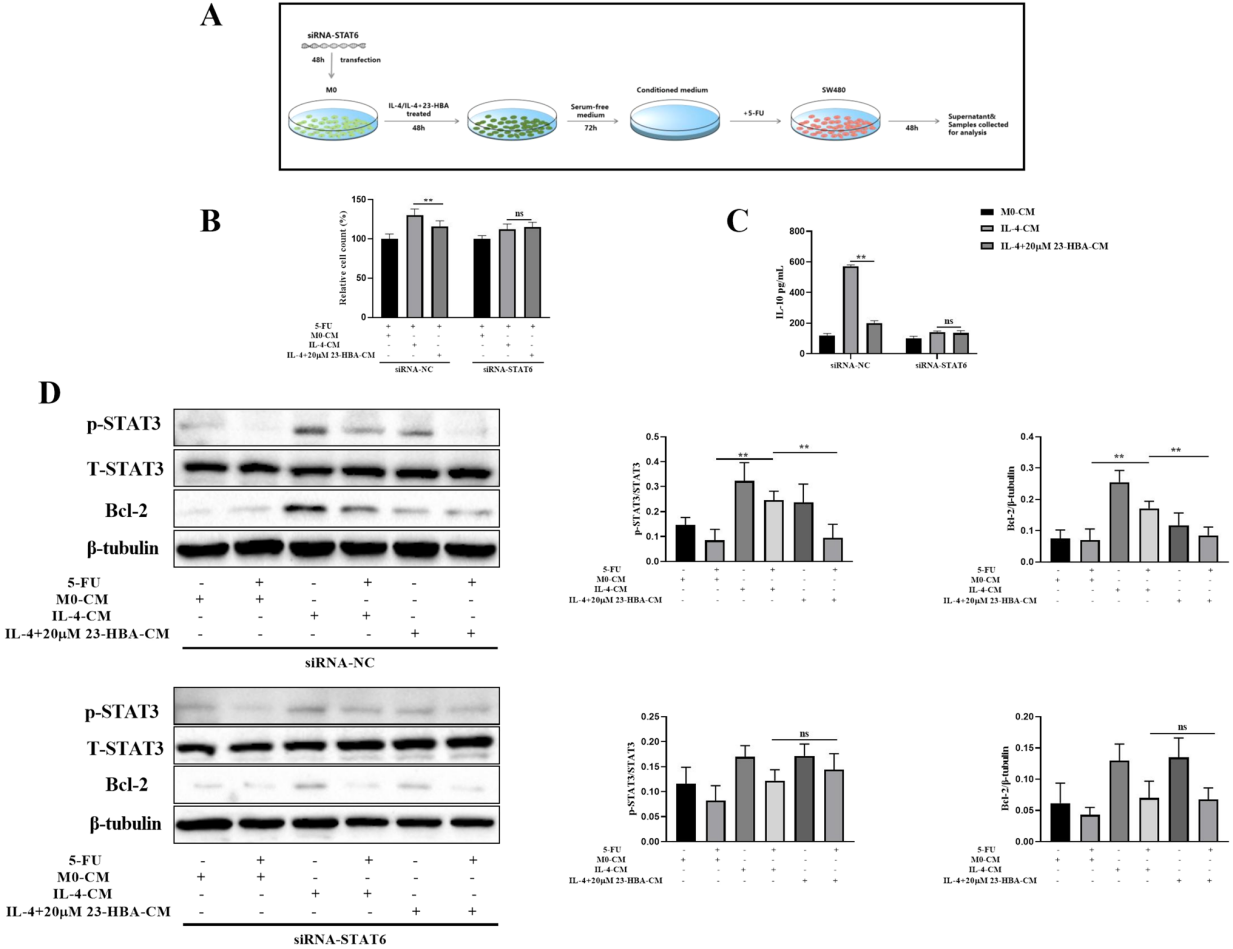
23-HBA downregulated M2 macrophage-induced 5-FU resistance of SW480 cells in a STAT6-dependent manner. **A** Schematic diagram of experiment. After transfecting macrophages with siRNA-NC and siRNA-STAT6, SW480 cells were treated with 5-FU (45 µM) for 48 h in M0-CM, IL-4-CM, and IL-4+20 µM 23-HBA-CM. **B** CCK8 assay was used to measure the relative number of SW480 cells. **C** IL-10 levels in conditioned medium were determined by ELISA. **D** The relative protein levels of p-STAT3/STAT3 and the expression of Bcl-2 protein were analyzed by western blot. β-tubulin was used as internal control. Data were presented as mean ± SD (*n* = 3). Statistical significance: *p < 0.05, **p < 0.01, *n.s* no significance

We performed additional research to determine if 23-HBA could reduce 5-FU resistance by inhibiting the polarization of M2 macrophages via STAT6 signaling. Upon transfecting macrophages with siRNA-NC (non-targeting control siRNA), it was observed that SW480 cells exposed to IL-4+23-HBA-CM showed significantly reduced resistance to 5-FU compared to IL-4-CM (Fig. 6B). This effect was accompanied by a decrease in the levels of IL-10 in IL-4-CM (Fig. 6C) and inhibition of the STAT3/Bcl-2 signaling pathway of SW480 cells (Fig. 6D). These findings were consistent with our observations in wild-type STAT6 macrophages. Interestingly, upon transfecting macrophages with siRNA-STAT6, we noticed that the series of reactions triggered by IL-4-induced M2 macrophages in SW480 cells did not appear to be significantly affected by 23-HBA administration (Fig. 6B–D). In general, the findings suggest that 23-HBA decreased M2 macrophage-mediated 5-FU resistance of SW480 cells through a STAT6-dependent mechanism.

### 23-HBA suppressed M2 macrophage polarization and attenuated colorectal cancer chemoresistance in vivo

**Fig. 7:**
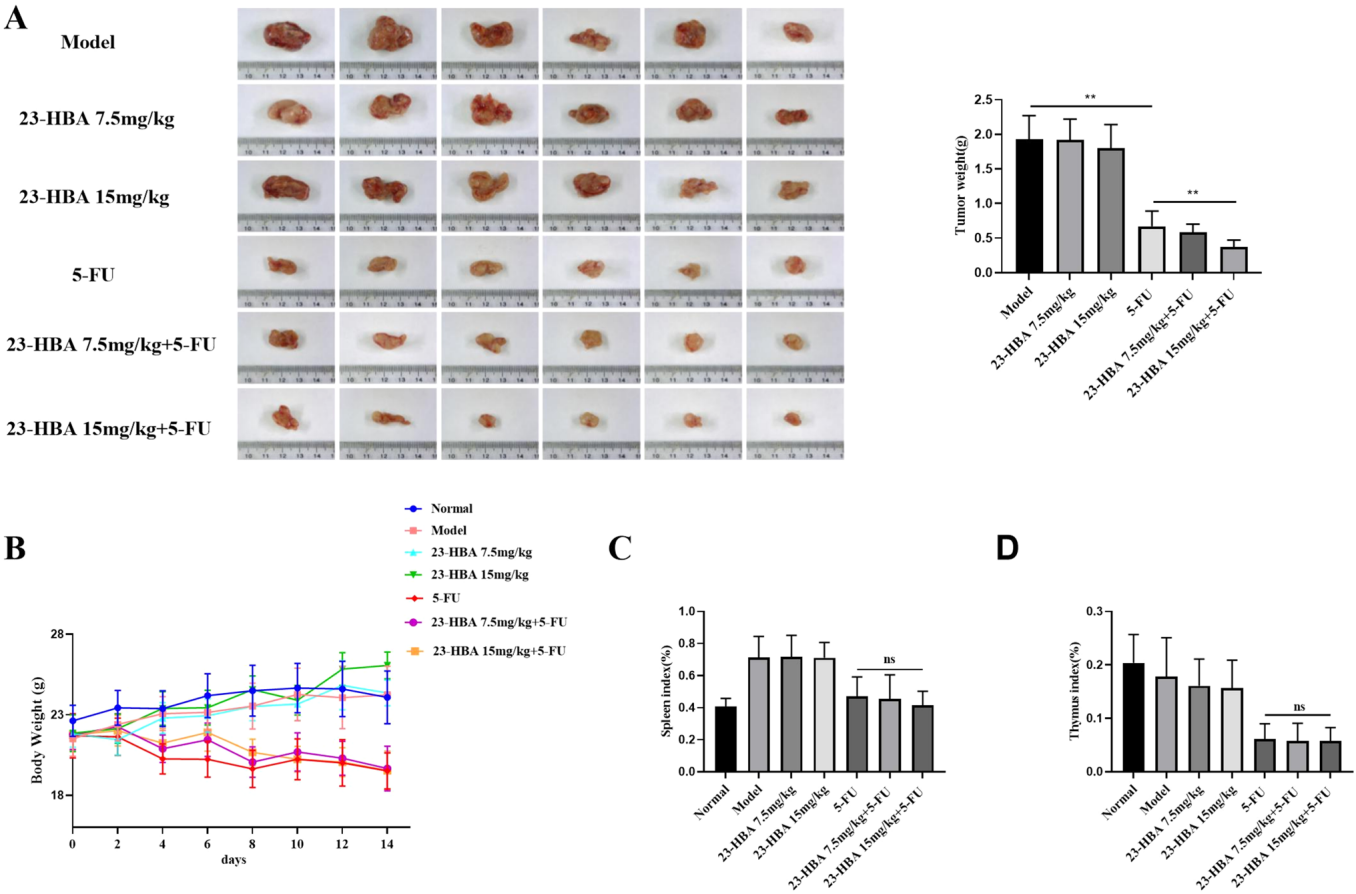
23-HBA attenuated tumor chemoresistance in vivo. **A** The picture of isolated tumors and the weight of tumors. **B** Body weight of mice in each group. **C** Spleen index. **D** Thymus index. Statistical significance: *p < 0.05, **p < 0.01, *n.s* no significance

**Fig. 8.**
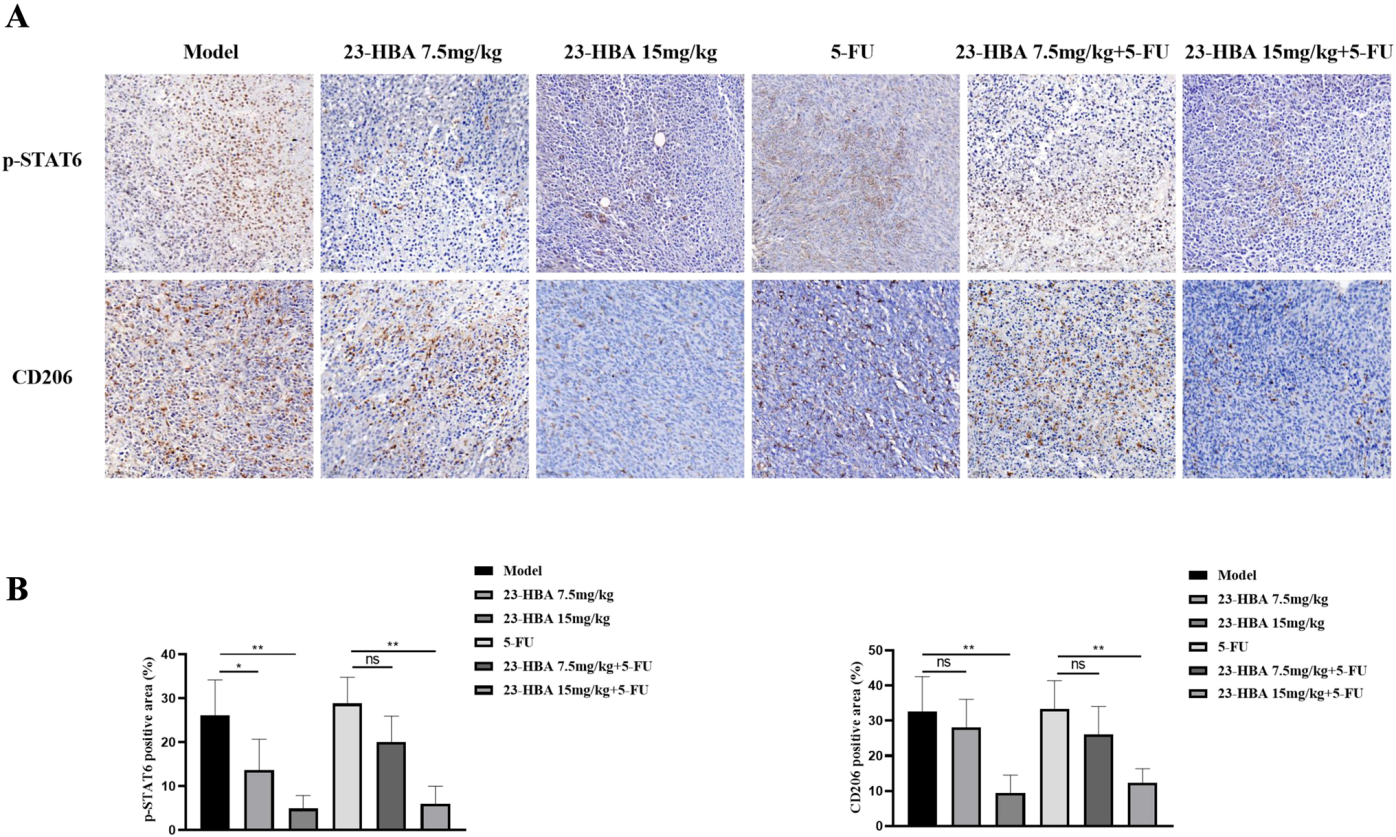
23-HBA inhibited M2 macrophage polarization via STAT6 in vivo. **A** p-STAT6 and CD206 staining of mice tumor tissues, Original magnification was 200×, bars represent 50 μm. **B** Quantification of immunohistochemistry staining of p-STAT6 and CD206. Data were presented as mean ± SD (*n* = 3). Statistical significance: *p < 0.05, **p < 0.01, *n.s* no significance

To validate our in vitro findings, we conducted in vivo experiments to examine the effect of 23-HBA on 5-FU resistance in colorectal cancer. In CT26 colorectal cancer mouse model, 23-HBA (7.5, 15 mg/kg) treatment slightly reduced tumor weight (Fig. 7A). It is worth noting that the co-treatment with 23-HBA (15 mg/kg) and 5-FU (25 mg/kg) effectively suppressed tumor growth, resulting in an approximate 80% reduction in tumor weight. This suggests that 23-HBA has the ability to augment the anti-cancer efficacy of 5-FU in vivo. In contrast, there was no significant difference in body weight, thymus index, or spleen index between mice in the 5-FU group and the combined group (Fig. 7B–D). Considering the effects of 23-HBA on the chemotherapy response of colorectal cancer mouse, we assume that 23-HBA may attenuate 5-FU resistance by modulating the macrophage phenotype. Immunohistochemistry staining in these tumors confirmed the inhibitory effects of 23-HBA on the expression of p-STAT6 and CD206 (Fig. 8). Additionally, we observed a notable reduction in the levels of IL-10 mRNA and Bcl-2 expression in the combined group compared to the 5-FU group (Fig. S1). Together, these findings indicate that 23-HBA may attenuate 5-FU resistance of colorectal cancer by inhibiting M2 macrophage polarization in vivo.

## Discussion

5-FU resistance is a huge challenge in improving the survival rate of patients with colorectal cancer [2]. The increasing amount of evidence suggests that M2 macrophages play a crucial role in promoting the development of 5-FU resistance in various malignancies, including colorectal cancer [29, 32–34]. Thus, blocking M2 polarization could present a potential strategy to overcome 5-FU resistance in colorectal cancer [35–37]. In this study, we demonstrated that exposing colorectal cancer SW480 cells to M2-CM notably enhanced their ability to resist apoptosis, which was correlated with a substantial increase in resistance to 5-FU. However, this resistance was mitigated by 23-HBA. Furthermore, 23-HBA reduced the proportion of M2 macrophages in tumor tissue, simultaneously enhancing the anti-cancer efficacy of 5-FU. These results can be largely attributed to the inhibitory impact of 23-HBA on the polarization of M2 macrophages.

Natural products have garnered increasing attention as potential adjunctive anti-tumor therapies in preclinical and clinical trials for their minimal toxicity, restricted side effects, and favorable tolerance properties [38]. Of note, they also regulate cytokine secretion and the expression of cell surface molecules, which contribute to maintain a balanced tumor microenvironment, especially by influencing the activation and polarization of macrophages [39]. 23-HBA is an effective active ingredient derived from the dried root of *P. chinensis*, known for its well-established anti-tumor effects. Our study adds another dimension to 23-HBA as an anti-tumor adjunctive drug. We discovered that 23-HBA effectively inhibited M2 macrophage polarization. Specifically, the administration of 23-HBA (20 µM) resulted in a significant reduction in the expression of the M2-specific marker CD206 and M2-associated genes. These findings highlight the potential of 23-HBA as an effective modifier with immunomodulatory properties.

The essential involvement of STAT6 in regulating the polarization of M2 macrophages induced by IL-4 or IL-13 has been extensively reported [20]. In our study, we observed robust phosphorylation of STAT6 protein in response to IL-4 stimulation. However, treatment with 23-HBA effectively inhibited STAT6 protein phosphorylation. The JAK2 protein has been reported to be activated and phosphorylated upon IL-4 binding to its receptors, which leads to the recruitment of STAT6 and its phosphorylation [21, 22]. Interestingly, we found that the phosphorylation of JAK2 protein induced by IL-4 remained unaffected by 23-HBA, suggesting that 23-HBA inhibited the phosphorylation of STAT6 protein through a JAK2-independent pathway.

M2 macrophages have been implicated in promoting chemoresistance, as activated macrophages with an M2 phenotype can protect cancer cells from the cytotoxic effects of chemotherapy by secreting cytokines that inhibit cell death signaling pathways [7–10]. Conversely, depleting M2 macrophages or inhibiting M2 phenotype polarization contributed to the improvement in therapeutic efficiency [9, 40–42]. In this study, we hypothesized that 23-HBA could modulate STAT6 signaling to inhibit M2 macrophage polarization and further influence the response of cancer cells to 5-FU chemotherapy. Indeed, we discovered that 23-HBA decreased the phosphorylation level of STAT6 protein in M2 macrophages, accompanied by a decrease in M2-related cytokine IL-10 secretion and an inhibition of STAT3/Bcl-2 signaling of cancer cells, resulting in a reduction in 5-FU resistance. Our findings are supported by a study suggesting that the IL-10/STAT3/Bcl-2 signaling pathway is crucial for promoting M2 macrophage-mediated chemoresistance [43]. Notably, after knocking down STAT6 in macrophages, the expression of CD206 stimulated by IL-4 was not reduced by 23-HBA, and 23-HBA failed to inhibit the activation of the IL-10/STAT3/Bcl-2 signaling pathway induced by M2 macrophages and the resulting resistance to 5-FU in cancer cells. These results indicate that 23-HBA suppressed M2 macrophage polarization in a STAT6-dependent manner, subsequently inhibiting M2 macrophage-mediated 5-FU resistance.

In addition, our animal experiments demonstrated that 23-HBA (15 mg/kg) exhibited a synergistic effect with 5-FU in CT26 colorectal cancer mice without obvious toxicity. Furthermore, 23-HBA effectively reduced the expression of p-STAT6 and CD206 in the tumor tissue. Additionally, the mRNA levels of IL-10 and Bcl-2 expression in the combined treatment group were significantly decreased compared to 5-FU group. However, the precise mechanism by which 23-HBA enhances the anti-tumor activity of 5-FU in vivo requires further investigation. Therefore, additional experiments are warranted to elucidate this question.

## Conclusion

Our study demonstrated for the first time that 23-HBA effectively inhibited M2 macrophage polarization both in vitro and in vivo, resulting in a reduction in 5-FU resistance in colorectal cancer. Furthermore, we propose that the mechanism by which 23-HBA can overcome 5-FU resistance in colorectal cancer involves inhibition of M2 macrophage polarization via STAT6 signaling. In summary, 23-HBA emerges as a promising and safe drug candidate for attenuating 5-FU resistance in colorectal cancer.

### Supplementary Information

Below is the link to the electronic supplementary material.

Supplementary file1 (DOCX 131 KB)

## Data Availability

Data are available upon reasonable request.

## Abbreviations

23-HBA: 23-Hydroxybetulinic acid
5-FU: 5-Fluorouracil
Arg1: Arginase 1
CCL2: Chemokine ligand 2
CD206: Cluster of differentiation 206
CRC: Colorectal cancer
IL-10: Interleukin-10
IL-4/13: Interleukin-4/13
JAK2: Janus Kinase 2
PMA: Phorbol myristate acetate
STAT6/3: Signal transducer and activator of transcription 6/3
TAM: Tumor-associated macrophages
TME: The tumor microenvironment
