# Supplementary material for: 23-Hydroxybetulinic acid attenuates 5-fluorouracil resistance of colorectal cancer by modulating M2 macrophage polarization via STAT6 signaling
Source: Cancer Immunol Immunother. 2024 Mar 30;73(5):83. doi: 10.1007/s00262-024-03662-0 (PMC10981607; doi:10.1007/s00262-024-03662-0)
Supplement: Supplementary file 1 — Supplementary file1 (DOCX 131 KB) [file 262_2024_3662_MOESM1_ESM.docx]

Tab. S1 Primers sets used for quantitative real-time PCR.

| Gene | Primer | Primer Sequences (5′ → 3′) |
| --- | --- | --- |
| Human CD206 | CD206-F | ACTGGGCAGAAGGAGTAACC |
|  | CD206-R | AATCCTCCGGACATTTGGGT |
| Human Arg-1 | Arg-1-F | GTGGAAACTTGCATGGACAAC |
|  | Arg-1-R | AATCCTGGCACATCGGGAATC |
| Human IL-10 | IL-10-F | GGCGCTGTCATCGATTTCTT |
|  | IL-10-R | ATAGAGTCGCCACCCTGATG |
| Human CCL2 | CCL2-F | CCATGGACCACCTGGACAAGCA |
|  | CCL2-R | GGTGTCTGGGGAAAGCTAGGGG |
| Human GAPDH | GAPDH-F | CGGAGTCAACGGATTTGGTCGTAT |
|  | GAPDH-R | AGCCTTCTCCATGGTGGTGAAGAC |
| Mouse IL-10 | IL-10-F | AAACAACTCCTTGGAAAACCTCG |
|  | IL-10-R | TCCCCAATGGAAACAGCTTAAAC |
| Mouse β-Actin | β-Actin-F | ATGACCCAGATCATGTTTGA |
|  | β-Actin-R | TACGACCAGAGGCATACAG |


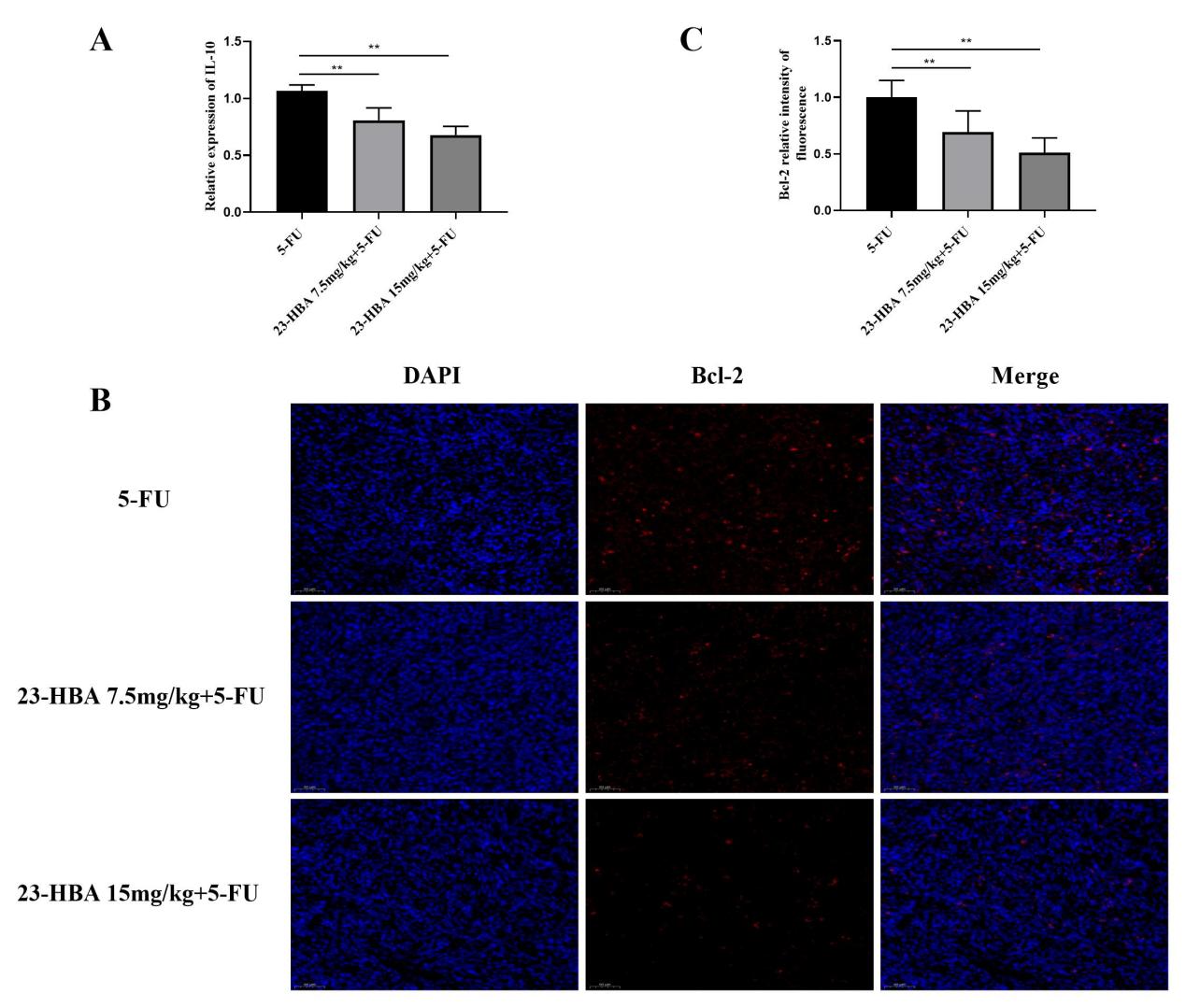
Fig. S1 23-HBA reduced tumor chemoresistance by downregulating Bcl-2 levels through IL-10 in vivo. **A** RT-PCR was performed to analyze the mRNA levels of IL-10 in tumors. The GAPDH gene was used as internal control. **B** Fluorescence staining of Bcl-2 in mice tumor tissues, Original magnification was 200×, bars represent 50 μm. **C** Quantification of the staining intensity of Bcl-2. Data were presented as mean ± SD (n = 3). Statistical signifcance: **p* < 0.05, ***p*<0.01
